# Preparation of Lignin-Based Carbon Materials and Its Application as a Sorbent

**DOI:** 10.3390/ma12071111

**Published:** 2019-04-03

**Authors:** Ling-Yan Meng, Ming-Guo Ma, Xing-Xiang Ji

**Affiliations:** 1 Engineering Research Center of Forestry Biomass Materials and Bioenergy, Beijing Key Laboratory of Lignocellulosic Chemistry, College of Materials Science and Technology, Beijing Forestry University, Beijing 100083, China; lilian1101@bjfu.edu.cn; 2 State Key Laboratory of Biobased material and Green papermaking, Qilu University of Technology (Shandong Academy of Sciences), Jinan 250353, China

**Keywords:** lignin, carbon, sorbent, preparation

## Abstract

The purpose of this article was to explore the influences of synthetic methods on the lignin-based carbon materials. In this paper, the lignin-based activated carbon materials were comparatively researched in ZnCl_2_ solution using various methods, including the microwave-assisted method, ultrasound method, and UV irradiation method, respectively. Scanning electron microscopy (SEM), Fourier transform infrared spectrometry (FT-IR), thermogravimetric analysis (TGA), and differential thermal analysis (DTA) were used to characterize the as-prepared samples. The effects of the synthetic parameters including the types of lignin, activated solution concentration, types of activated solution, and synthetic methods on the morphologies, thermal stability, and specific surface area of samples were comparatively investigated in detail. The specific surface area of lignin-based activated carbon increased to 473.8, 765.3, and 211.2 m^2^∙g^−1^ using the microwave-assisted method, ultrasound method, and UV irradiation method, respectively, compared with that of the control (113.4 m^2^∙g^−1^). The lignin-based carbon materials displayed the enhanced absorptive capacity, compared with that of the control. These novel synthetic methods reported here maybe have a guiding significance for the synthesis of carbon materials using the lignin as precursors.

## 1. Introduction

Recently, the carbon materials have received more attention due to their several advantages of light, stable, defect sites, and large surface area [1,2,3]. Natural biomass is an important precursor of carbon materials because of its abundant, renewable, and environmental friendly, compared with the non-renewable fossil resources of oil, coal, and gases [4,5]. There are many reports on the synthesis of carbon materials using natural biomass as precursor [6,7,8]. For example, Subramanian et al. obtained activated carbon with good electrochemical properties in neutral electrolyte from banana fibers using ZnCl_2_ and KOH [9]. Du et al. reported the preparation of activated carbon hollow fibers from ramie at low temperature for electric double-layer capacitor applications [10,11]. In the literature, microcrystalline cellulose, and cellulose nanocrystals were also applied as precursors of carbon composites materials by an ultrasound method combining calcination in ethylene glycol [12]. Wang et al. reviewed the recent development in engineered biochar productions and applications [13].

It is well known that biomass consisted of cellulose, hemicellulose, and lignin. Lignin is an abundant biopolymer with a high carbon content and high aromaticity, which is promising as a raw material for the fuel, chemicals, and lignocellulosic biopolymers [14]. More importantly, lignin is an excellent precursor for the preparation of high-value carbon materials [15]. Fu et al. employed black.

liquor lignin obtained from pulp and paper industry as the precursor for preparing activated carbon by physical activation with steam [16]. Mesoporous carbon was also synthesized from pre-cross-linked lignin gel impregnated with a surfactant as the pore-forming agent and then was activated through physical and chemical methods to obtain activated mesoporous carbon with specific surface area of 1148 m^2^∙g^−1^ and a pore volume of 1.0 cm^3^∙g^−1^ [17]. Recently, an amorphous carbon material with an amazing high carbon yield of 57% was achieved for sodium-ion batteries by utilizing the emulsification interaction between pitch and lignin to suppress the graphitization of pitch during the carbonization [18]. Zhang et al. used lignin-derived byproducts as precursors to construct an interconnected hierarchical porous nitrogen-doped carbon via hydrothermal treatment and activation [19]. 

Some successful methods were employed for the synthesis of carbon materials from lignin including the electrospinning [20,21], freeze-drying method [22], hydrothermal carbonization [23,24], carbonization [25,26], etc. In general, the synthetic methods played an important role in the microstructure and properties of lignin-based carbon materials. The synthetic methods such as the microwave and ultrasound method have been accepted as promising green technologies for the synthesis of functional materials [27,28,29]. In our previous work, the microwave-assisted treatment was used to synthesize the mesoporous carbon sponge as an efficient adsorbent for Cr(VI) removal from a supramolecular microcrystalline cellulose–polymer system [30]. However, as far as we know, the comparative study of lignin-based activated carbon materials using microwave, ultrasound, and UV irradiation method has not been reported yet. Microwave heating is a promising method in materials fields due to its unique effects, as compared with conventional heating, such as rapid volumetric heating, increased reaction rates and shortened reaction times, and energy saving. The ultrasound method is also a promising green method in the synthesis of functional materials due to intense local heating, high pressures, and extremely rapid cooling rates during the ultrasound agitation procedure.

Herein, we reported a comparatively research for synthesizing lignin-based carbon materials using the different synthetic parameters including the types of lignin, activated solution concentration, types of activated solution, and synthetic methods. The influences of synthetic methods such as the microwave-assisted method, ultrasound method, and UV irradiation method on the lignin-based carbon materials were explored and discussed in detail. This study could thus guide the development of various processing technologies for the high quality of lignin-based carbon materials. 

## 2. Experimental Section

### 2.1. Materials

All chemicals used in the sample preparation were of analytical grade and used as received without further purification. All experiments were conducted under air atmosphere with ionized water. Four types of lignin were used in this paper including Kraft lignin, hydrolysate lignin, alkali lignin, and Longlive lignin. Both Kraft lignin and hydrolysate lignin were obtained by Shandong Sun Paper Industry Joint Stock Co., Ltd. Jining, PR China, alkali lignin was supplied by Geyi Energy Company in Hefei, Anhui province of PR China, meanwhile Longlive lignin were obtained from Shandong Longlive Bio-technology Co., Ltd. Dezhou, PR China. Both Kraft lignin and hydrolysate lignin were produced from Poplar wood, alkali lignin was produced from the wheat straw, and Longlive lignin was produced from the corncob residue after hydrolysis of hemicelluloses [31]. As for the preparation of Longlive lignin, the corn-cob was treated hydrothermally to degrade the hemicelluloses and obtain xylo-oligosaccharides. Then, the residue was treated with an alkaline solution to release lignin. After that, the effluent, following alkaline treatment, was adjusted to acidic condition to precipitate the lignin.

### 2.2. Synthetic Process of Lignin-Based Carbon Materials

4.00 g lignin was added into the 40 wt % ZnCl_2_ solution (20 mL) under vigorous stirring for 2 h. The product was separated from the solution by centrifugation, and used as precursor to be calcinated at 700 °C for 3 h with a heating rate of 5 °C∙min^-1^ under the protection of flowing nitrogen gas in the tube furnace. The as-obtained sample was used as control. For comparison, 4.00 g Longlive lignin was added into the 40 wt % KOH, 40 wt % NaOH, and 40 wt % H_3_PO_4_ (20 mL), respectively, under vigorous stirring for 2 h. The product was separated from the solution by centrifugation, and used as precursor to be calcinated at 700 °C for 3 h with a heating rate of 5 °C∙min^−1^ under the protection of flowing nitrogen gas in the tube furnace.

Moreover, the 40 wt % ZnCl_2_ solution (20 mL) with 4.00 g lignin was heated in a microwave oven (MDS-6G, Sineo, Shanghai, PR China) at 700 W for 30 min. After being cooled down naturally to room temperature, the products were separated from the solution by centrifugation, and used as precursors to be calcinated to 700 °C for 3 h with a heating rate of 5 °C∙min^−1^ under the protection of flowing nitrogen gas in the tube furnace. The as-obtained lignin-based carbon materials were used for further characterizations. For comparison, the 40 wt % ZnCl_2_ solution (20 mL) with 4.00 g lignin was subjected to sonication (Xin-Zhi, JY92-2D, Shanghai, PR China) at air condition for 30 min in the pulse mode, while other reaction conditions were the same. Furthermore, the 40 wt % ZnCl_2_ solution (20 mL) with 4.00 g lignin was treated with UV irradiation for 30 min, while other reaction conditions were the same.

### 2.3. Characterization

Fourier transform infrared (FT-IR, Karlsruhe, Germany) spectroscopic measurements were carried out on Bruker VERTEX 70V spectrophotometer. Scanning electron microscopy (SEM, Hitachi, Tokyo, Japan) images were recorded with Hitachi 3400 N, all samples were Au coated prior to examination by SEM. Samples were deposited on thin amorphous carbon films supported by copper grids from ultrasonically processed ethanol solutions. The thermogravimetry analysis (TGA, Shimadzu, Japan) curves were measured on Shimadzu DTG-60 with a heating rate of 10 °C∙min^−1^ under nitrogen atmosphere, and each sample was weighed between 3 and 5 mg for analysis. The specific Brunauer–Emmett–Teller surface areas were measured on a Quantachrome Quadrasorb Station 1 by nitrogen adsorption at 77.3 K and the pore-size distributions were measured on a Quantachrome Quadrasorb Station 1 by Barrett–Joyner–Halenda pore size desorption isotherms at 77.3 K.

The adsorption experiments were carried out on a shaker at 200 rpm. Typically, 50 mL of methylene blue (MB) solution with desired concentration and 20 mg of different lignin-based carbon materials were added into 100 mL glass flasks. Then, the samples were collected by centrifugation after different time intervals to measure the concentration of MB by UV–vis spectra (Shimadzu UV2450, Shimadzu, Japan).

## 3. Results and Discussion

### 3.1. The Influences of Four Types of Lignin

**Figure 1:**
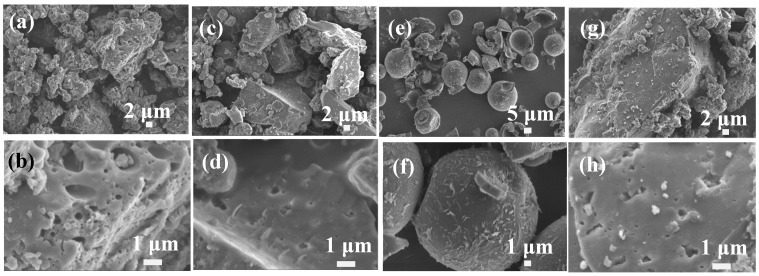
SEM images of four typical types of lignin including (**a,b**) Kraft lignin, (**c,d**) hydrolysate lignin, (**e,f**) alkali lignin, and (**g,h**) Longlive lignin.

In this paper, four typical types of lignin were used as precursors to synthesize the lignin-based carbon materials including Kraft lignin, hydrolysate lignin, alkali lignin, and Longlive lignin. As shown in Figure 1, one can see these four typical types of lignin with different shapes. The Kraft lignin has irregular morphologies with most big and small holes (Figure 1a,b). Hydrolysate lignin displayed block-like shape with some small pores (Figure 1c,d). However, the alkali lignin exhibited completely different shapes of microspheres with several micrometers (Figure 1e,f). Most sheet-like shape dispersed on the surface of the microspheres. Longlive lignin, which is similar with Kraft lignin, had the irregular morphologies with most holes (Figure 1g,h). It is easy to obtain the industry scale Longlive lignin and favor the development of high-value applications of industry wastes. Therefore, Longlive lignin was chosen as a precursor to synthesize the lignin-based carbon materials. 

**Figure 2:**
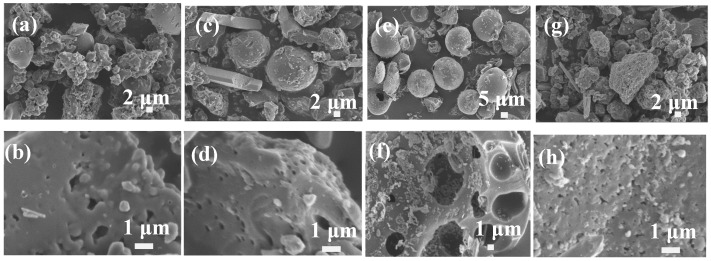
SEM images of lignin-based carbon materials in 40 wt% ZnCl_2_ solution using four typical types as precursors: (**a,b**) Kraft lignin, (**c,d**) hydrolysate lignin, (**e,f**) alkali lignin, and (**g,h**) Longlive lignin.

The lignin-based carbon materials were obtained in 40 wt % ZnCl_2_ solution by calcination using four typical types of precursors, as shown in Figure 2. The shapes of four typical types of lignin did not greatly change. The lignin-based carbon materials have smooth surface using Kraft lignin as precursor (Figure 2a,b), compared with that of the Kraft lignin. The pores were not clearly observed due to the melt of particles. As for the lignin-based carbon materials using hydrolysate lignin as precursor, the pores appeared (Figure 2c,d). The microspheres broken using alkali lignin as precursor (Figure 2e,f). Both the hollow microspheres and the inside hollow microspheres were observed in Figure 2f. When the Longlive lignin was used as precursor, the lignin-based carbon materials had a loose structure (Figure 2g,h). It observed pores among these particles. Based on these results, the types of lignin influenced the microstructure of lignin-based carbon materials. In the literature, Shi et al. reported the impact of three types of lignin by various extraction methods on the microstructure and mechanical properties of lignin-based carbon fibers [32]. Obviously, these results were similar with the previous results. In this paper, four typical types of precursors had effects on the microstructure of lignin-based carbon materials. Choosing appropriate lignin as precursor is important for the applications of lignin-based carbon materials.

### 3.2. The Influences of The Activated Solution Concentration

**Figure 3:**
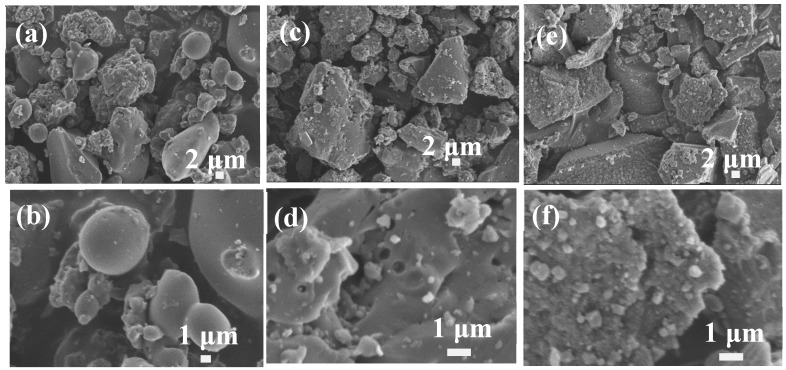
SEM images of lignin-based carbon materials using Longlive lignin as precursor in different ZnCl_2_ solution: (**a,b**) 10 wt %, (**c,d**) 20 wt %, and (**e,f**) 60 wt %.

Du et al. investigated the effect of ZnCl_2_ impregnation concentration on the microstructure and electrical performance of ramie-based activated carbon hollow fiber using ramie as precursor at low temperature for electric double-layer capacitor applications [12]. It reported that the morphology and pore structure development of ramie-based activated carbon hollow-fiber depend greatly on ZnCl_2_ concentration because ZnCl_2_ solution not only can swell and dissolve cellulose but also can serve as skeleton of newborn pores. In this paper, the influences of the ZnCl_2_ activated solution concentration on the lignin-based carbon materials were investigated in detail. As shown in Figure 2, it obtained the lignin-based carbon materials with loose structure consisted of particles using Longlive lignin as precursor in 40 wt % ZnCl_2_ solution. When the ZnCl_2_ solution decreased from 40 wt % to 10 wt %, lignin-based carbon materials had the aggregation irregular shape consisted of particles and some smooth surface (Figure 3a,b). When the ZnCl_2_ solution decreased from 40 wt % to 20 wt %, it observed the irregular shape with some pores for the lignin-based carbon materials (Figure 3c,d). However, when the ZnCl_2_ solution increased from 40 wt % to 60 wt %, the lignin-based carbon materials displayed irregular shape (Figure 3e,f). Interestingly, the particles and spheres dispersed at the surface of the lignin-based carbon materials. In the literature, it was reported that high ZnCl_2_ solution swell and dissolve cellulose [10,11], having an adverse impact on the microstructure of lignin-based carbon materials. Considering the experimental results, 40 wt % ZnCl_2_ solution was chosen as the activated solution concentration so as to obtain the lignin-based carbon materials with ideal shapes and structures.

### 3.3. The Influences of The Types of Activated Solution

**Figure 4:**
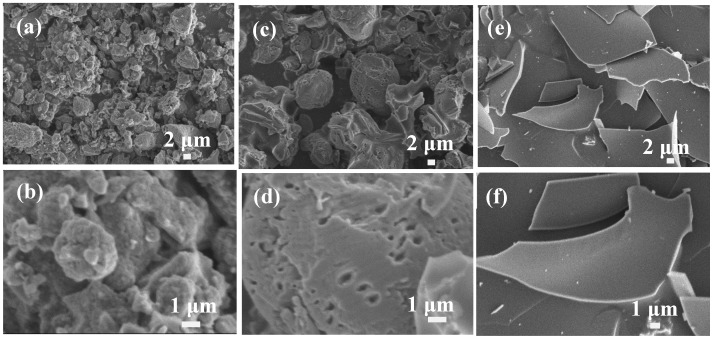
SEM images of lignin-based carbon materials using Longlive lignin as precursor in different types of activated solution: (**a,b**) 40 wt % KOH, (**c,d**) 40 wt % NaOH, and (**e,f**) 40 wt % H_3_PO_4_.

In addition to ZnCl_2_ solution, all the KOH, NaOH, and H_3_PO_4_ were also applied as activated solution to change the microstructure and property of lignin-based carbon materials. We also investigated the influences of the types of activated solution including KOH, NaOH, and H_3_PO_4_ on the shapes of lignin-based carbon materials using Longlive lignin as precursor, as shown in Figure 4. In the 40 wt % KOH solution, the lignin-based carbon materials have loose structure (Figure 4a,b), which are similar with that of ZnCl_2_ solution. In the 40 wt % NaOH solution, regular pores were observed at the surface of lignin-based carbon materials (Figure 4c,d). Using the 40 wt % H_3_PO_4_ solution, a completely different shape of big sheets with smooth surface were obtained for the lignin-based carbon materials (Figure 4e,f). These results demonstrated that the types of activated solution played an important role in the shape, structure, and pore of lignin-based carbon materials.

### 3.4. The Influences of Synthetic Methods

**Figure 5:**
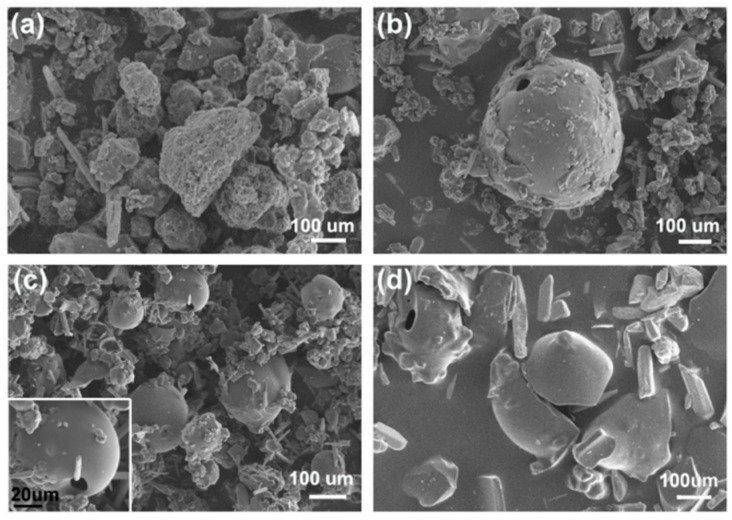
SEM images of (**a**) control and (**b-d**) lignin-based carbon materials in 40 wt % ZnCl_2_ solution using (**b**) microwave, (**c**) ultrasound, and (**d**) UV irradiation, respectively.

The shape and microstructure of the as-prepared lignin-based carbon materials were characterized with SEM. Figure 5 showed the SEM images of control and lignin-based carbon materials in 40 wt % ZnCl_2_ solution using microwave, ultrasound, and UV irradiation, respectively. Obviously, it observed the irregular shape for the control (Figure 5a). However, lignin-based carbon materials displayed the hollow microspheres and irregular shape using the microwave-assisted method (Figure 5b). Using the ultrasound irradiation instead of the microwave-assisted method, large numbers of hollow microspheres with smooth surface were obtained (Figure 5c). Using the UV irradiation method, most of the hollow microspheres with smooth surface broke (Figure 5d). These results indicated that the synthetic methods had an effect on the shape and microstructure of the as-prepared lignin-based carbon materials. In the literature, the carbon spheres were obtained after the hydrothermal carbonization of glucose followed by pyrolysis [33] and activated carbon hollow fibers with pore structure were prepared by one-step activation process and calcination of ramie at ZnCl_2_ solution [10,11]. There was also report about the impact of three types of lignin by various extraction methods on microstructure and mechanical properties of lignin-based carbon fibers [30]. In general, many parameters such as the types of lignin, treatment methods, carbonization temperature, carbonization time, activated solution, etc, had an effect on the structure and property of lignin-based carbon materials [13,31,32]. In this paper, the experimental results further indicated the influences of the synthetic methods on the lignin-based carbon materials. It may be due to the different mechanism among these synthetic methods.

**Figure 6:**
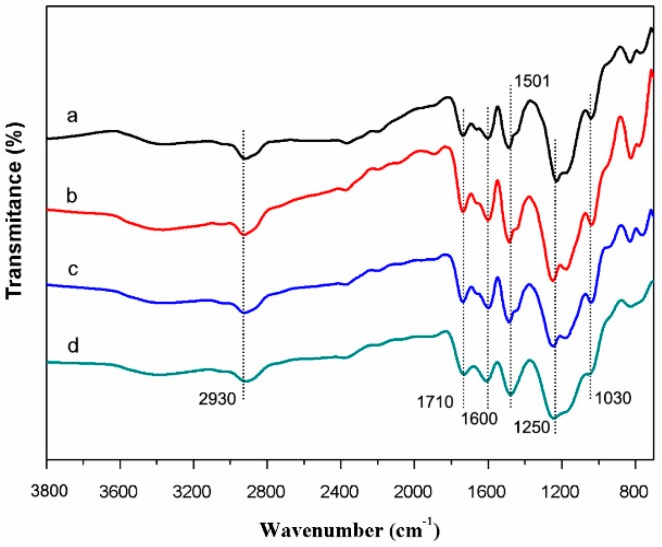
FT-IR spectra of (**a**) control and (**b-d**) precursors in 40 wt % ZnCl_2_ solution using (**b**) microwave, (**c**) ultrasound, and (**d**) UV irradiation, respectively.

FT-IR spectra were widely applied to further study the functional groups of samples. Figure 6 showed the FT-IR spectra of lignin-based carbon materials in 40 wt % ZnCl_2_ solution using the microwave-assisted, ultrasound, and UV irradiation, respectively. All the samples displayed similar FT-IR peaks. The bands at 2930 cm^−1^ was assigned to the C-H asymmetric and symmetrical vibrations in methyl and methylene groups. The peak at ~1600 and 1501 cm^−1^ indicated the aromatic skeletal vibration of lignin. The peak at 1250 cm^−1^ is due to guaiacyl ring breathing with C=O stretching [34]. 

**Figure 7:**
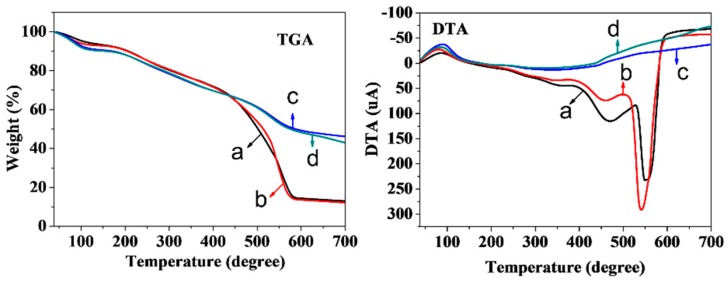
TGA and DTA curves of (**d**) control and (**a–c**) precursors in 40 wt% ZnCl_2_ solution using(a) ultrasound, (**b**) microwave, and (**c**) UV irradiation, respectively.

The thermal behavior of the control and precursors was investigated with TGA and DTA under nitrogen atmosphere, as shown in Figure 7. The sample with UV irradiation displayed similar TGA curve, compared with that of the control (Figure 7c,d). Meanwhile, both the samples with the microwave-assisted method and ultrasound irradiation method exhibited similar TGA curves (Figure 7a,b). The total weight losses of the control and lignin-based carbon materials using UV irradiation, ultrasound, and microwave are 53.0%, 57.0%, 85.0%, and 85.3%, respectively. The first degradation stage was at ~100 °C, indicating the removal of the absorbed water. The second degradation stage was mainly due to the slow carbonization of lignin at 200~450 °C [35]. The last degradation stage appeared at 500~600 °C. It observed endothermic peaks at 455 and 540 °C in the DTA curves with the microwave-assisted method, and 470 and 555 °C with the ultrasound irradiation method. These results indicated that both the microwave-assisted method and ultrasound irradiation method influenced the thermal behavior of lignin-based carbon materials. However, the UV irradiation method had a slight effect on the thermal behavior of the sample.

**Figure 8:**
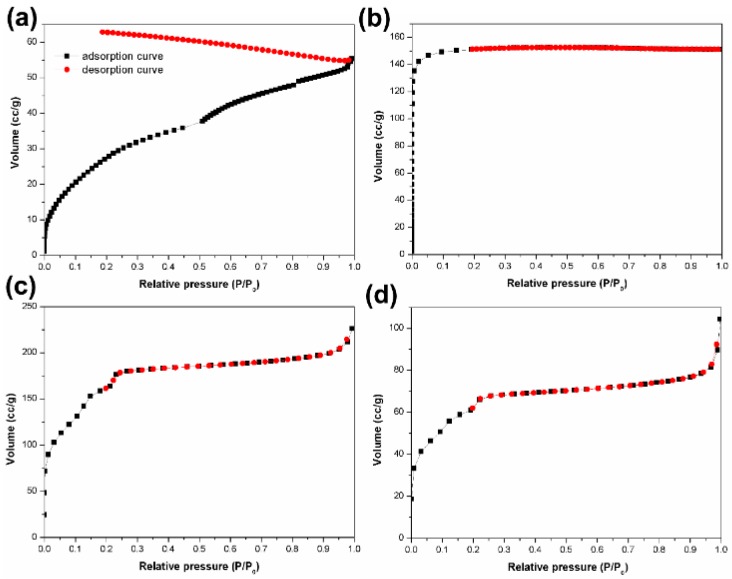
N_2_ adsorption isotherms of the (**a**) control and (**b–d**) lignin-based carbon materials in 40 wt % ZnCl_2_ solution using (**b**) microwave, (**c**) ultrasound, and (**d**) UV irradiation, respectively.

The specific surface area is important for the applications of the lignin-based carbon materials. It obtained the specific surface area of 113.4 m^2^∙g^−1^ for control (Figure 8a). However, the values of specific surface area increased to 473.8 m^2^∙g^−1^, 765.3 m^2^∙g^−1^, and 211.2 m^2^∙g^−1^ using the microwave-assisted, ultrasound, and UV irradiation method, respectively (Figure 8b-d). Based on the results of specific surface area, both samples using microwave-assisted method and ultrasound irradiation method exhibited the increase specific surface area, further confirming their influences on the lignin-based carbon materials. Similarly, it observed the slight increase specific surface area of the sample via UV irradiation method, compared with that of the control. Du et al. obtained activated carbon with high specific surface area of 1178 m^2^∙g^−1^ [10], which is much higher than that of this paper. In Du’s work, the samples were washed in 1 mol L^−1^ HCl solution. However, the samples of this article did not post-treated in acid solution, inducing the relatively low specific surface area. In general, the treatment in acid solution had an important influence in the formation of carbon materials with high specific surface area by the removal of the surface organic matter and the trace alkaline soluble salt [36]. For example, Sarkar et al. prepared the activated carbon with Brunauer-Emmett-Teller (BET) surface area of 1063 m^2^∙g^-1^ by phosphoric acid (H_3_PO_4_) activation of potassium hydroxide (KOH) pulping spent liquor lignin from rice straw and compared with KOH hydroxide activation at 800 °C for 60 min at an impregnation ratio of 2.5 [37]. Moreover, the treatment in acid solution changed the structure and pore size of carbon materials, and increased the reaction step during the preparation of carbon materials. In this paper, it is expected that carbon materials were achieved with improved properties without post-treatment.

**Figure 9:**
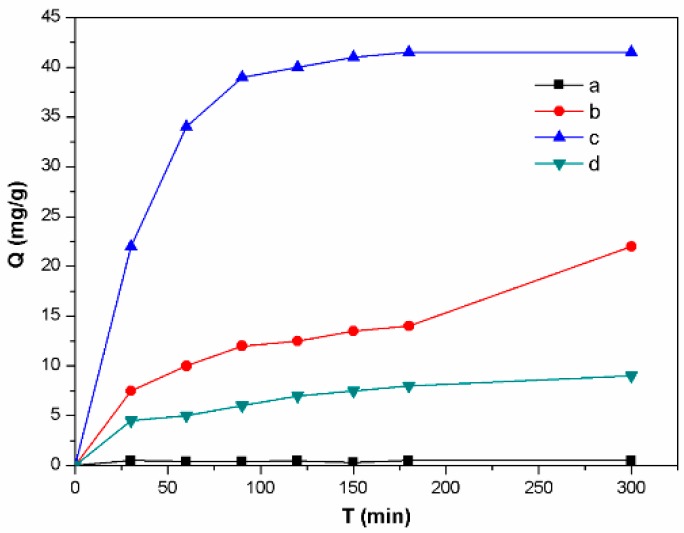
The adsorption rates of the (**a**) control and (**b–d**) lignin-based carbon materials in 40 wt % ZnCl_2_ solution using (**b**) microwave, (**c**) ultrasound, and (**d**) UV irradiation, respectively.

The adsorption experiment was performed to evaluate the adsorption ability of lignin-based carbon materials in the dark by UV-vis spectrum. Figure 9 showed the adsorption rate curves of methylene blue on the control and lignin-based carbon materials samples in 40 wt% ZnCl_2_ solution using the microwave-assisted, ultrasound, and UV irradiation, respectively. As for the control, no obvious adsorption property was observed (Figure 9a). However, all the lignin-based carbon materials samples synthesized using the microwave-assisted, ultrasound, and UV irradiation displayed the increase adsorption property. It obtained the adsorption capacity of 24 mg∙g^-1^, 43 mg∙g^-1^, and 10 mg∙g^−1^ for the lignin-based carbon materials samples synthesized using the microwave-assisted, ultrasound, and UV irradiation, respectively (Figure 9b–d). These results indicated that the as-obtained lignin-based carbon materials displayed the enhanced absorptive capacity. Based on the results of specific surface area, the increase adsorption ability of lignin-based carbon materials was due to the increase of specific surface area, compared with that of the control. In the previous report, Liu et al. synthesized the carbon/iron oxide composites, which displayed the adsorption capacity of about 20–51 mg∙g^-1^ for methylene blue [38]. In comparison with that in the literature, it obtained the relatively low adsorption capacity for the lignin-based carbon materials. However, the adsorption capacity of lignin-based carbon materials greatly increased, compared with that of control, indicating the influences of synthetic methods on the lignin-based carbon materials. 

## 4. Conclusions

In summary, lignin-based carbon materials were synthesized in ZnCl_2_ solution using the microwave-assisted, ultrasound, and UV irradiation method, respectively. The influences of the synthetic parameters including the types of lignin, activated solution concentration, types of activated solution, and synthetic methods on the morphologies, thermal stability, and specific surface area of samples were comparatively investigated in detail. The synthetic methods played an important role in the morphologies, thermal stability, specific surface area, and adsorption ability of lignin-based carbon materials. It obtained the high value of specific surface area of 765.3 m^2^∙g^−1^ and the adsorption capacity of 43 mg∙g^−1^ using the ultrasound method. This lignin-based carbon materials have promising applications in the dye removal and wastewater treatment fields. This synthetic strategy reported here may open a new way to synthesize other lignin-based carbon materials. 

## References

[1] Cao S.W., Yu J.G. (2016). Carbon-based H_2_-production photocatalytic materials. J. Photochem. Photobiol. C Photochem. Rev..

[2] Hou H.S., Qiu X.Q., Wei W.F., Zhang Y., Ji X.B. (2017). Carbon anode materials for advanced sodium-ion batteries. Adv. Energy Mater..

[3] Wang J., Xu F., Jin H.Y., Chen Y.Q., Wang Y. (2017). Non-noble metal-based carbon composites in hydrogen evolution reaction: Fundamentals to applications. Adv. Mater..

[4] Biswal M., Banerjee A., Deo M., Ogale S. (2016). From dead leaves to high energy density supercapacitors. Energy Environ. Sci..

[5] He X.J., Li R.C., Qiu J.S., Xie K., Ling P.H., Yu M.X., Zhang X.Y., Zheng M.D. (2012). Synthesis of mesoporous carbons for supercapacitors from coal tar pitch by coupling microwave-assisted KOH activation with a MgO template. Carbon.

[6] Zhai Y.P., Dou Y.Q., Zhao D.Y., Fulvio P.F., Mayes R.T., Dai S. (2011). Carbon materials for chemical capacitive energy storage. Adv. Mater..

[7] Qu W.H., Xu Y.Y., Lu A.H., Zhang X.Q., Li W.C. (2015). Converting biowaste corncob residue into high value added porous carbon for supercapacitor electrodes. Bioresour. Technol..

[8] Luan Y.T., Wang L., Guo S.E., Jiang B.J., Zhao D.D., Yan H.J., Tian C.G., Fu H.G. (2015). A hierarchical porous carbon material from a loofah sponge network for high performance supercapacitors. RSC Adv..

[9] Subramanian V., Luo C., Stephan C., Nahm A.M., Thomas K.S., Wei B.Q. (2007). Supercapacitors from activated carbon derived from banana fibers. J. Phys. Chem. C.

[10] Du X., Zhao W., Wang Y., Wang C.Y., Chen M.M., Qi T., Hua C., Ma M.G. (2013). Preparation of activated carbon hollow fibers from ramie at low temperature for electric double-layer capacitor applications. Bioresour. Technol..

[11] Du X., Zhao W., Ma S.H., Ma M.G., Qi T., Wang Y., Hua C. (2016). Effect of ZnCl_2_ impregnation concentration on the microstructure and electrical performance of ramie-based activated carbon hollow fiber. Ionics.

[12] Liu S., Liu Y.J., Deng F., Ma M.G., Bian J. (2015). Comparison of the effects of microcrystalline cellulose and cellulose nanocrystals on the Fe_3_O_4_/C nanocomposites. RSC Adv..

[13] Wang B., Gao B., Fang J. (2017). Recent advances in engineered biochar productions and applications. Crit. Rev. Environ. Sci. Technol..

[14] Rinaldi R., Jastrzebski R., Clough M.T., Ralph J., Kennema M., Bruijnincx P.C.A., Weckhuysen B.M. (2016). Paving the way for lignin valorisation: Recent advances in bioengineering, biorefining and catalysis. Angew. Chem.Int. Ed..

[15] Baker D.A., Rials T.G. (2013). Recent advances in low-cost carbon fiber manufacture from lignin. J. Appl. Polym. Sci..

[16] Fu K.F., Yue Q.Y., Gao B.Y., Sun Y.Y., Zhu L.J. (2013). Preparation, characterization and application of lignin-based activated carbon from black liquor lignin by steam activation. Chem. Eng. J..

[17] Saha D., Li Y.C., Bi Z.H., Chen J.H., Keum J.K., Hensley D.K., Grappe H.A., Meyer H.M., Dai S., Paranthaman M.P. (2014). Studies on supercapacitor electrode material from activated lignin-derived mesoporous carbon. Langmuir.

[18] Li Y.M., Hu Y.S., Li H., Chen L.Q., Huang X.J. (2016). A superior low-cost amorphous carbon anode made from pitch and lignin for sodium-ion batteries. J. Mater. Chem. A.

[19] Zhang L.M., You T.T., Zhou T., Zhou X., Xu F. (2016). Interconnected hierarchical porous carbon from lignin-derived byproducts of bioethanol production for ultra-high performance supercapacitors. ACS Appl. Mater. Interfaces.

[20] Zhao Y., Liu Y., Tong C.C., Ru J., Geng B.Y., Ma Z.Q., Liu H.Z., Wang L.K. (2018). Flexible lignin-derived electrospun carbon nanofiber mats as a highly efficient and binder-free counter electrode for dye-sensitized solar cells. J. Mater. Sci..

[21] Garcia-Mateos F.J., Berenguer R., Valero-Romero M.J., Rodriguez-Mirasol J., Cordero T. (2018). Phosphorus functionalization for the rapid preparation of highly nanoporous submicron-diameter carbon fibers by electrospinning of lignin solutions. J. Mater. Chem. A.

[22] Zeng Z.H., Wang C.X., Zhang Y.F., Wang P.Y., Seyed S., Seyed I., Pei Y.M., Chen M.J., Lu X.H. (2018). Ultralight and highly elastic graphene/lignin-derived carbon nanocomposite aerogels with ultrahigh electromagnetic interference shielding performance. ACS Appl. Mater. Interfaces.

[23] Correa C.R., Stollovsky M., Hehr T., Rauscher Y., Rolli B., Kruse A. (2017). Influence of the carbonization process on activated carbon properties from lignin and lignin-rich biomasses. ACS Sustain. Chem. Eng..

[24] Guo N.N., Li M., Sun X.K., Wang F., Yang R. (2017). Enzymatic hydrolysis lignin derived hierarchical porous carbon for supercapacitors in ionic liquids with high power and energy densities. Green Chem..

[25] Liu W.S., Yao Y.M., Fu O.L., Jiang S.H., Fang Y.C., Wei Y., Lu X.H. (2017). Lignin-derived carbon nanosheets for high-capacitance supercapacitors. RSC Adv..

[26] Pan Z.Z., Dong L.B., Lv W., Zheng D.Q., Li Z.J., Luo C., Zheng C., Yang Q.H., Kang F.Y. (2017). A hollow spherical carbon derived from the spray drying of corncob lignin for high-rate-performance supercapacitors. Chem. Asian J..

[27] Zhu Y.J., Chen F. (2014). Microwave-assisted preparation of inorganic nanostructures in liquid phase. Chem. Rev..

[28] Meng L.Y., Wang B., Ma M.G., Lin K.L. (2016). The progress of microwave-assisted hydrothermal method in the synthesis of functional nanomaterials. Mater. Today Chem..

[29] Bang J.H., Suslick K.S. (2010). Applications of ultrasound to the synthesis of nanostructured materials. Adv. Mater..

[30] Liu Y.J., Liu S., Li Z.W., Ma M.G., Wang B. (2018). Microwave synthetic mesoporous carbon sponge as an efficient adsorbent for Cr(VI) removal. RSC Adv..

[31] Yang S., Wen J.L., Yuan T.Q., Sun R.C. (2014). Characterization and phenolation of biorefinery technical lignins for lignin–phenol–formaldehyde resin adhesive synthesis. RSC Adv..

[32] Shi X.J., Wang X., Tang B., Dai Z., Chen K.F., Zhou J.H. (2018). Impact of lignin extraction methods on microstructure and mechanical properties of lignin-based carbon fibers. J. Appl. Polym. Sci..

[33] Wang J., Shen L., Ding B., Nie P., Deng H.F., Dou H., Zhang X.G. (2014). Fabrication of porous carbon spheres for high-performance electrochemical capacitors. RSC Adv..

[34] Yuan T.Q., Sun S.N., Xu F., Sun R.C. (2011). Isolation and physico-chemical characterization of lignins from ultrasound irradiated fast-growing poplar wood. BioResources.

[35] Yang H.P., Yan R., Chen H.P., Zheng C.G., Lee D.H., Liang D.T. (2006). In-depth investigation of biomass pyrolysis based on three major components: Hemicellulose, cellulose and lignin. Energy Fuels.

[36] Frank E., Steudle L.M., Ingildeev D., Sporl J.M., Buchmeiser M.R. (2014). Carbon fibers: Precursor systems, processing, structure, and properties. Angew. Chem.Int. Ed..

[37] Sarkar M., Tian C., Jahan M.S. (2018). Activated carbon from potassium hydroxide spent liquor lignin using phosphoric acid. TAPPI J..

[38] Liu S., Yao K., Fu L.H., Ma M.G. (2016). Selective synthesis of Fe_3_O_4_, γ-Fe_2_O_3_, and α-Fe_2_O_3_ using cellulose-based composites as precursors. RSC Adv..

